# Combinatorial Effect of PLK1 Inhibition with Temozolomide and Radiation in Glioblastoma

**DOI:** 10.3390/cancers13205114

**Published:** 2021-10-12

**Authors:** Arvind Pandey, Satyendra C. Tripathi, Junhua Mai, Samir M. Hanash, Haifa Shen, Sankar Mitra, Robert C. Rostomily

**Affiliations:** 1 Department of Neurosurgery, Houston Methodist Research Institute, Houston, TX 77030, USA; 2 Department of Radiation Oncology, Houston Methodist Research Institute, Houston, TX 77030, USA; smitra2@houstonmethodist.org; 3 Department of Biochemistry, All India Institute of Medical Sciences, Nagpur 440003, India; sctripathi@aiimsnagpur.edu.in; 4 Department of Clinical Cancer Prevention, University of Texas M. D. Anderson Cancer Center, Houston, TX 77030, USA; shanash@mdanderson.org; 5 Department of Nanomedicine, Houston Methodist Research Institute, Houston, TX 77030, USA; jmai@houstonmethodist.org (J.M.); hshen@houstonmethodist.org (H.S.)

**Keywords:** glioblastoma, side population, PLK1 inhibitor, volasertib, temozolomide, radiation

## Abstract

**Simple Summary:**

There is a critical need to identify readily translatable adjuncts to potentiate the dismal median survivals of only 15–20 months in glioblastoma (GBM) patients after standard of care, i.e., concurrent Temozolomide (TMZ) and radiation (XRT) therapy. Here we demonstrated that the Polo-like kinase 1 (PLK1) inhibitor volasertib, which has been employed in cancer clinical trials, has activity against GBM in the contexts of both as monotherapy and as an adjunct to standard of care (SOC). In addition to corroborating the known effects of volasertib, we found novel impacts of volasertib on mitochondrial membrane potential, ROS generation, persistent DNA damage and signaling pathways such as ERK/MAPK, AMPK and glucocorticoid receptor. Together these studies support the potential importance of PLK1 inhibitors as an adjunct to GBM SOC therapy that warrants further preclinical investigation.

**Abstract:**

New strategies that improve median survivals of only ~15–20 months for glioblastoma (GBM) with the current standard of care (SOC) which is concurrent temozolomide (TMZ) and radiation (XRT) treatment are urgently needed. Inhibition of polo-like kinase 1 (PLK1), a multifunctional cell cycle regulator, overexpressed in GBM has shown therapeutic promise but has never been tested in the context of SOC. Therefore, we examined the mechanistic and therapeutic impact of PLK1 specific inhibitor (volasertib) alone and in combination with TMZ and/or XRT on GBM cells. We quantified the effects of volasertib alone and in combination with TMZ and/or XRT on GBM cell cytotoxicity/apoptosis, mitochondrial membrane potential (MtMP), reactive oxygen species (ROS), cell cycle, stemness, DNA damage, DNA repair genes, cellular signaling and in-vivo tumor growth. Volasertib alone and in combination with TMZ and/or XRT promoted apoptotic cell death, altered MtMP, increased ROS and G2/M cell cycle arrest. Combined volasertib and TMZ treatment reduced side population (SP) indicating activity against GBM stem-like cells. Volasertib combinatorial treatment also significantly increased DNA damage and reduced cell survival by inhibition of DNA repair gene expression and modulation of ERK/MAPK, AMPK and glucocorticoid receptor signaling. Finally, as observed in-vitro, combined volasertib and TMZ treatment resulted in synergistic inhibition of tumor growth in-vivo. Together these results identify new mechanisms of action for volasertib that provide a strong rationale for further investigation of PLK1 inhibition as an adjunct to current GBM SOC therapy.

## 1. Introduction

Glioblastoma (GBM) is the most lethal and primary malignant brain tumor with median survivals of 15–20 months [1,2]. Introduced in 2005, concurrent temozolomide (TMZ) chemotherapy and radiation (XRT) remain the current standard of care (SOC) treatment for GBM [3]. This paradigm increased median survival from ~12 to 15 months compared with XRT alone [3] while the addition of tumor treating fields improves median survivals from 16 to 20.9 months [4]. These incremental improvements underscore the urgent need to identify new targeted approaches to potentiate genotoxic TMZ/XRT SOC therapy [5]. To accelerate this process, drugs under active clinical investigation in other cancers with demonstrated preclinical activity in GBM could be rapidly repurposed for clinical trials to establish complementary or synergistic interactions with TMZ/XRT. We, therefore, investigated the activity and mechanisms of action of one such candidate, volasertib, a selective polo-like kinase 1 (PLK1) inhibitor, as a targeted adjunct to potentiate SOC therapy in GBM.

PLK1 is a well-characterized member of the polo-like kinase family that functions primarily through cell cycle regulation [6]. High PLK1 expression levels in many cancers including GBM are associated with poor outcomes [7]. PLK1 interaction with DNA damage response during mitosis [8] and PLK1 inhibition-dependent cell cycle arrest, apoptosis and tumor regression [9] implicate it as a mediator in the crosstalk between cellular signaling and DNA damage and repair. Previous preclinical studies in GBM demonstrated an anti-tumor role of PLK1 inhibition either with TMZ [2,10] or with radiation only [11]. However, the potential therapeutic effects of volasertib, an ATP-competitive PLK1 targeted inhibitor [12] in the clinically relevant context of concurrent TMZ and XRT have not yet been addressed.

Therefore, we characterized the functional consequences and mechanisms of action of PLK1 inhibition alone and in combination with TMZ and XRT in GBM cells. In this study, we provided unique evidence that volasertib alone or in combination with TMZ and/or XRT potentiated cytotoxic responses and exhibited significantly higher anti-tumor responses in conjunction with diverse functional effects on mitochondrial membrane potential (MtMP), ROS, cell cycle, DNA damage, stemness and oncogenic signaling pathways such as ERK/MAPK, AMPK activity and glucocorticoid receptor. Overall, our data identified novel mechanisms of action for volasertib and supported the potential clinical relevance of PLK1 inhibition as an adjunct to current GBM therapy.

## 2. Materials and Methods

### 2.1. Cell Culture and Reagents

Glioblastoma cell lines LN229 (ATCC CRL-2611), T98G (ATCC CRL-1690) and U87MG (ATCC HTB-14) were procured from ATCC, USA and maintained in recommended culture condition. Patient-derived glioblastoma cell line BT115 was obtained from Drs. David Baskin and Martyn A. Sharpe, Department of Neurosurgery, Houston Methodist Hospital, TX, USA. Parental BT115 cells were maintained in DMEM/F12 supplemented with 5% FBS and 1% penicillin/streptomycin mixture. CD133+ tumor sphere (TS) cells were maintained and cultured in ultra-low attachment plates (Corning, NY, USA) with Cancer Stem Premium media (Cat. 20141-500, ProMab Biotechnologies, Inc. Richmond, CA, USA). All cell lines were authenticated and routinely tested for mycoplasma contamination.

### 2.2. Cell Viability (MTT Assay)

Volasertib and temozolomide were purchased from Selleckchem, Houston, TX, USA. The cytotoxic effects of volasertib (nM) (Selleckchem Cat: S2235) alone and with TMZ (µM) (Selleckchem Cat: S1237) in GBM cells were determined by MTT assay as described earlier [13]. A detailed method has been provided as a Supplementary Methodology. The combination index was calculated by CompuSyn software for drug combinations and general dose-effect analysis developed by Ting-Chao Chou, Memorial Sloan-Keltering Cancer Center, New York, NY, USA and Nick Martin Massachusetts Institute of Technology, Cambridge, MA, USA [14].

### 2.3. Irradiation of GBM Cells

RS 2000 X-Ray Irradiator was used for irradiating GBM cells. Based on the clonogenicity of cells, 3 Gy of XRT was used for clonogenic assessment). For the cellular response, 5 Gy of radiation was given to cells just before drug treatment as concurrent treatment.

### 2.4. Flow Cytometry

After the 24 h of treatment, cells were analyzed by flow cytometry using the BD LSRII and BD FACS Fortessa. Single-cell suspensions were prepared by Accutase (Millipore-Sigma, St. Louis, MO, USA) treatment and mechanical dissociation and stained in 1× PBS + 0.2 mM EDTA + 0.5% BSA. Specific kits were used for detection of Apoptosis (BD Pharmingen, Cat:556547, San Diego, CA, United States.), Mitochondrial membrane potential (Abcam, Waltham, MA, USA; Cat: ab113852), Cellular ROS (Invitrogen, Waltham, MA, USA; Ref: C10492), Cell cycle (Abcam, Cat: ab139418) and CD133 staining (Miltenyi Biotec, Cat: 130-113-108, Bergisch Gladbach, Germany) as per manufacturer instructions. G0-G1 transition assay for quiescent cells was performed as described earlier [15]. Data obtained was analyzed by FCS express V6 and FlowJo software (BD, USA).

### 2.5. ROS Assay

The cells were treated with TMZ, volasertib and combination with radiation (5 Gy) for 24 h. The single-cell suspension was prepared at 10^6^ cells/mL. CellRox Green (Invitrogen, Ref: C10492, Massachusetts, MA, USA) was added at a final concentration of 750 nM and incubated for 60 min at 37 °C, 5% CO_2_, protected from light. The samples were analyzed by Flow cytometry and data was acquired on the GFP channel.

### 2.6. Tumor Sphere (TS) Formation

After the 24 h of treatment, single live cell (1 × 10^4^) obtained by treatment with Accutase (Millipore-Sigma) were cultured in ultra-low attachment plates (Corning) with Cancer Stem Premium media (ProMab Cat. 20141-500) and routinely examined microscopically for sphere formation. On day 10, the number of the spheres (containing more than 50 cells) were counted and microscopic images (Evos FL Auto, Life Technology, CA, USA) were captured at 10× magnification. Sphere forming efficiency was calculated as the number of spheres formed per the total number of cells seeded.

### 2.7. Side Population Analysis

Assessment of side population was performed by labeling Hoechst 33342 in the absence or presence of Fumitremorgin C (FTC), an inhibitor of ABCG2 as described previously [16,17].

### 2.8. Human Phosphokinase Array

The Human Phospho-Kinase was performed after cell lysate preparation from GBM cells using Human Phospho-Kinase Array Kit (R&D Systems, Minneapolis, MN, USA; Cat: ARY003B) as per manufacturer’s instructions [18]. The original figures have been provided as Supplementary Figure S5.

### 2.9. Immunoblotting

The cell lysate was prepared using Complete Lysis-M (Roche, Basel, Switzerland) solution containing a cocktail of proteinase inhibitors and phosphatase inhibitors. The protein concentration was determined using the BCA protein assay (Pierce Chemical, Dallas, TX, USA). Samples were subjected to 4% to 20% Mini-PROTEAN TGX (BioRad, Hercules CA, USA) PAGE and transferred to PVDF membranes using Trans-Blot Turbo Transfer System (BioRad). The blots were probed with primary antibodies [STAT1 (CST, Cat: CST-14994, Danvers, MA, USA), phosphoSTAT1 (CST, Cat: CST-9167), Akt (CST, Cat: CST-9272), phosphoAkt (CST, Cat: CST-4060), p38α MAPK (CST, Cat: CST-9228), phospho-p38α MAPK (CST, Cat: CST-4511), phospho-mTOR (CST, Cat: CST-5536) and β-Actin (CST, Cat: CST-3700)] and then incubated with horseradish peroxidase-linked secondary anti-rabbit or anti-mouse antibodies (GE, Piscataway, NJ, USA). Original figures of western blot have been included as Supplementary Figure S5 and antibodies dilutions have been provided as Supplementary Table S1.

### 2.10. Neutral Comet Assay

The Comet analysis of DNA double-strand breaks was carried out at neutral pH using Trevigen comet assay kit (Cat: 4250-050-K, Gaithersburg, MD, USA) following the manufacturer’s protocol. The image was analyzed by the Open Comet plugin in ImageJ for various comet parameters [19]. A detailed method has been provided as a Supplementary Methodology.

### 2.11. Clonogenic Cell Survival Analysis

LN229 and BT115 cells were treated with TMZ, volasertib and combination with and without 3Gy of radiation for 24 h. The cells were detached using Trypsin-EDTA (0.25%) and 1000 live cells from each sample were plated in triplicate in 6 well plates. After 14 days, the colonies were stained with 0.5% crystal violet solution in 50% methanol. Clonogenic efficiency was measured by % area and/or % intensity through colony area (ImageJ) [20].

### 2.12. RT2 Profiler PCR Array

LN229 cells were collected after 24 h of treatment followed by the release of drugs for additional 24 h. RNA was extracted by RNeasy plus mini kit (Qiagen, Cat:74134, Hilden, Germany) and cDNA was prepared by RT2 First Strand Kit (Qiagen, Cat:33041). cDNA was analyzed by real-time PCR using the RT2 SYBR Green qPCR Mastermix (Qiagen, Cat:330500) and DNA Repair kit (Qiagen, Cat: PAHS-042Z). The analysis was performed as per the manufacturer’s instructions.

### 2.13. Xenograft Models and Treatment

LN229 cells (1 × 10^6^) were injected subcutaneously into the dorsal flank of athymic BALB/c nude mice (4-week-old, female) from Jackson laboratory. Once the tumor volume reached 100 mm^3^, animals were divided into five subgroups: control 1 (TMZ vehicle), control 2 (volasertib vehicle), TMZ (Selleckchem, Cat: S1237) volasertib (Selleckchem, Cat: S2235), combination (TMZ + volasertib) (*n* = 3 mice). TMZ (25 mg/kg formulated in 10% DMSO in sterile PBS) and administrated intravenously every 3 days. TMZ (25 mg/kg formulated in 10% DMSO in sterile PBS) and administrated intravenously every 3 days. Volasertib was formulated in hydrochloric acid (0.1 N) and diluted with 0.9% NaCl at 10 mg/kg concentration and delivered intravenously every 3 days. Each mouse’s tumor size was measured every 3 days after the first injection by a Vernier caliper. The volume of the tumor was calculated with the formula: volume = (length × width^2^)/2. In this case, 30 days after the injection, the mice were euthanized and the tumors were dissected for histological analysis.

### 2.14. Immunohistochemistry Analysis

The paraffin-fixed sections from xenografts were deparaffinized followed by graded rehydration. After antigen retrieval (citrate buffer, pH 6.0) and blocking in 5% serum, the slides were incubated with the primary antibodies overnight at 4 °C. The immunohistochemical staining was performed using Ki67 (Abcam- Cat: 15580), survivin (CST, Cat: 2808), tunel, cleaved caspase-3 (CST, Cat: 9661), gH2AX (Abcam, Cat: 81299), CD133 (Abcam, Cat: 19898) and CD73 (CST, Cat: 13160). Staining was quantified by the IHC profiler plugin in ImageJ software. Data obtained was present as violin plot with the data point and automatic pairwise comparison was used to check significant level between groups.

### 2.15. Statistical Analysis

The data analysis was performed using the statistical software GraphPad Prism (GraphPad Software Version 8.0). Statistical significance of the difference between the two test groups was analyzed by the Student’s *t*-test and multiple comparisons were assessed by one-way or two-way ANOVA as appropriate. Significance levels were set at *p* ≤ 0.05.

## 3. Results

### 3.1. PLK1 Inhibition Promotes Apoptotic Cell Death and Reduces Cell Survival Alone and in Combination with TMZ and Radiation

**Figure 1:**
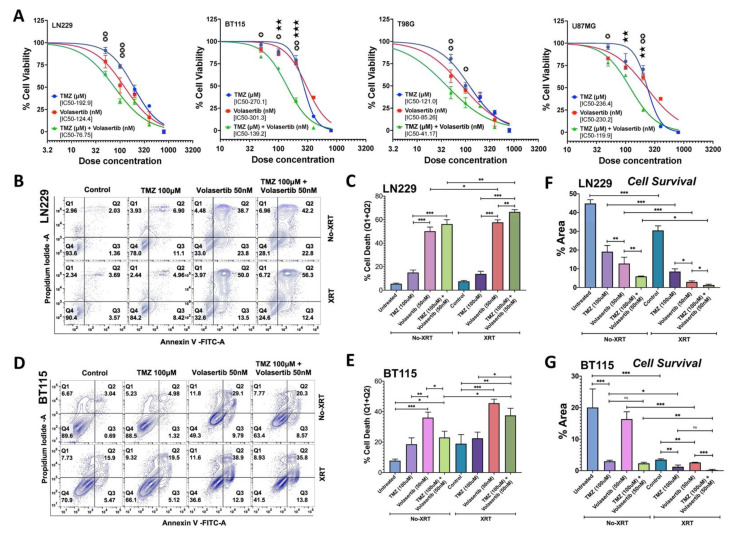
Cytotoxic effect of volasertib with TMZ and radiation on cell survival. Cell viability was measured by MTT assay after TMZ (µM), volasertib (nM) and its combination (µM: nM) treatment at the various dose of 50, 100, 200, 400 and 800 in LN229, BT115, U87MG and T98G cells for 24 h The graph presented as Normalize of Transform X of dose vs. response (✪ vs. volasertib, ★ vs. TMZ). Two-way ANOVA with Tukey’s multiple comparison test, error bars, SD; (significance at ≤0.05) (**A**) Apoptotic cell death was measured by Annexin V-FITC and PI staining after treatment of TMZ (100 µM), volasertib (50 nM) and its combination with or without radiation (XRT-5Gy) in LN229 cells (**B**) and BT115 cells (**D**). Graphical representation of % cell death (Q1 + Q2) from three different experiments LN229 cells (**C**) and in BT115 cells (**E**). One-way ANOVA, error bars, SD; (significance at ≤0.05). Quantitation of Clonogenic assessment after 24 h of drug and radiation treatment in LN229 cells (**F**) and BT115 cells (**G**). Quantitation of clonogenicity was performed from three experiments using % area covered by cells analyzed by colony area plugin of Image J. One-way ANOVA and unpaired *t*-test, error bars, SD; [significance at ≤0.05 (*), ≤0.002 (**), ≤0.001 (***) and 0.12 (ns)].

We first examined the combined effect of volasertib and TMZ on cell viability using commercial glioblastoma cell lines LN229, U87MG, T98G and patient-derived (BT115) glioblastoma cell lines. Cell viability was measured after 24 h of treatment with various doses of TMZ (50, 100, 200, 400 and 800 µM), volasertib (50, 100, 200, 400 and 800 nM) and combination at 1000:1 ratio of TMZ and volasertib. The combined treatment of volasertib and TMZ synergistically reduced cell viability in a dose-dependent manner with IC50 values of 76.75, 41.17, 119.9 and 139.2 for LN229, T98G, U87MG and BT115 cells, respectively. BT115 cells were more resistant to TMZ and volasertib compared to commercial cell lines tested (Figure 1A). The combined effect of two drugs was analyzed by drug combination index (CI) value at IC50 which was 0.221, 0.233, 0.203 and 0.480 for BT115, LN229, U87 and T98G, respectively, suggesting a synergistic effect (CI < 1.0) when combined. Based on moderate IC50 value and combination index (CI) value, we selected LN229 and BT115 to evaluate the additional cytotoxic effect with radiation treatment. The cells were irradiated (5Gy) just before the treatment of a sub-lethal dose of volasertib (50 nM), TMZ (100 µM), and its combination to measure apoptotic cell death.

We observed that volasertib treatment significantly increased cell death compared to TMZ treatment. Radiation treatment further increases the cell death caused by TMZ, volasertib and their combined treatment. In the LN229 cells, combined treatment of TMZ and volasertib showed a marginal increase over volasertib alone (*P*-0.09). However, radiation treatment significantly increased the effect of volasertib alone (50.22% to 57.7%, *P*-0.03) and combination treatment (56.28% to 66.53%, *P*-0.005) compared to non-irradiated cells. The cytotoxic effect of combined treatment was significantly high in irradiated cells (Figure 1B,C). In contrast, volasertib alone was more effective than combinatorial drug treatment (*P*-0.04) in BT115 cells. Radiation significantly increased the effect of combined drug treatment (23.02% to 37.52%, *P*-0.02) and marginal increase the effect of volasertib alone (36.05 to 45.53%, *P*-0.12) in BT115 cells (Figure 1D,E). These data indicate that radiation potentiated the cytotoxic effect of volasertib alone and its combination with TMZ in a cell line-dependent manner.

To complement cell survival assessment, we also performed a clonogenic assay (Supplementary Figure S1) which provides unique insight into reproductive capacity as opposed to short-term toxicity. Results in LN229 showed congruent increases in cell death and reduced clonogenic cell survival (Figure 1C,F). However, for BT115 the same assays showed divergent results for TMZ and volasertib mediated cell death and clonogenic survival (Figure 1E,G). This result, while surprising, may indicate that BT115 has intrinsic properties that manifest in an assay-dependent fashion under unique pharmacologic stresses of volasertib (cell cycle inhibition) and TMZ (alkylation) which were not apparent with the addition of radiation (Figure 1G).

### 3.2. Volasertib Drives Concerted Disruption of MtMP, Activation of ROS and G2-M Arrest in GBM Cells without the Transition to the Quiescent State

**Figure 2:**
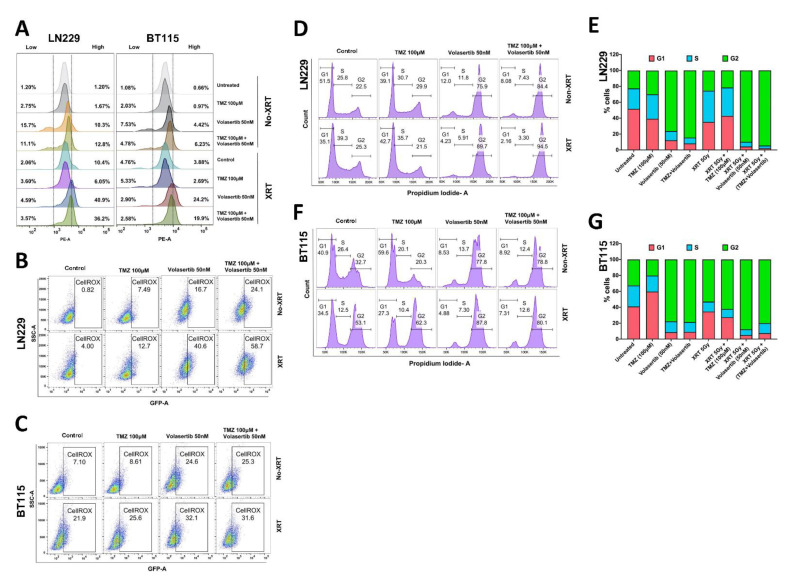
Effect of volasertib and its combination with TMZ and Radiation (XRT-5Gy) on MtMP, ROS and cell cycle by Flow Cytometry. Mitochondrial membrane potential was measured by TMRE (tetramethylrhodamine, ethyl ester) staining in LN229 and BT115 cells and quantified the percentage of cells with decreased (low) or increased (high) MtMP in controls and treated cells (**A**) Cellular ROS measured after 24 h of treatment by CellRox Green staining in LN229 (**B**) and BT115 cells (**C**). Cell cycle analysis by propidium iodide staining in LN229 cells (**D**,**E**) and BT115 cells (**F**,**G**).

As deregulation of MtMP is associated with an increase in cellular ROS and apoptosis [21,22], we first measured the alterations in MtMP. We observed higher MtMP in irradiated LN229 and BT115 cells after volasertib alone (40.9% and 24.2% in LN229 and BT115, respectively) or combined treatment with TMZ (36.2% and 19.9% in LN229 and BT115, respectively). Non-irradiated cells showed an increase in disrupted (low and high both) MtMP while irradiated cells specifically showed high MtMP after volasertib alone and combined treatment with TMZ (Figure 2A). In general, the volasertib and combined treatment disrupted the MtMP which was further significantly potentiated by radiation treatment. The combined treatment showed a higher effect on MtMP compared to TMZ alone but was found less effective compared to the volasertib-only treatment.

Next, we measured cellular ROS and as expected, intercellular ROS was increased in irradiated cells compared to non-irradiated cells. In LN229 cells, a significant increase in ROS was observed after treatment with volasertib treatment alone (16.7%) and in combination with TMZ (24.1%) which was further potentiated by radiation to 40.6% in volasertib and 58.7% in combination treatment (Figure 2B). However, volasertib alone treatment significantly increased ROS (24.6%) which was potentiated by radiation (32.1%) in BT115 cells (Figure 2C). Collectively, these data demonstrated increases in ROS after treatment with volasertib alone or in combination with TMZ, which further potentiated by radiation treatment depending upon GBM cell types.

Since increased MtMP disruption and ROS production lead to cell cycle arrest [23], we analyzed cell cycle distribution. We observed markedly increased G2-M arrest after volasertib treatment (75.9%) as well as in combination with TMZ (84.4%) as compared to control G2-M level in LN229 cells (22.5%). With the addition of radiation, G2-M arrest was further increased in volasertib alone (75.9% to 89.7%) and combination with TMZ (84.4% to 94.5%) (Figure 2D,E). In BT115 cell, volasertib treatment alone effectively caused G2-M arrest to 77.8% from 32.7% in control cells which was further potentiated to 87.8% by radiation treatment. We did not see any significant change in G2-M arrest after combinatorial drug and radiation treatment in BT115 cells (Figure 2F,G).

### 3.3. Inhibition of PLK1 Reduces Cancer Cell Stemness and Abrogates Side Population (SP) in GBM Cells

**Figure 3:**
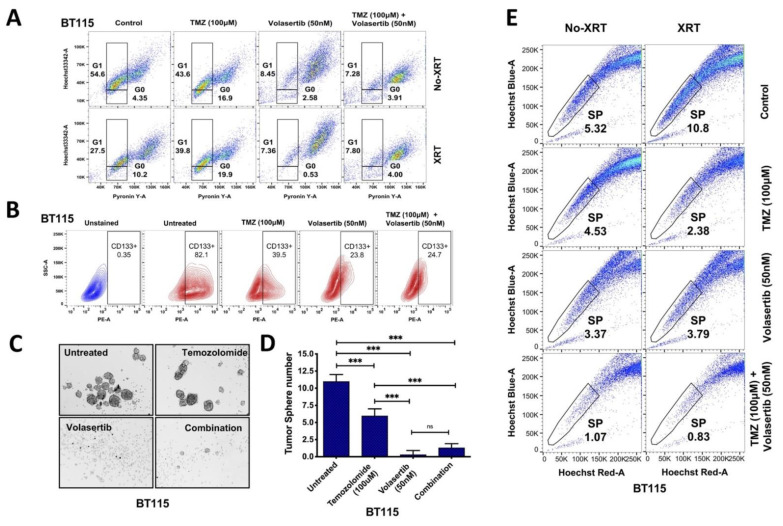
The treatment of volasertib and its combination reduced CD133 cancer stem cell marker, tumorsphere formation and side-population in BT115 cells. G1–G0 transition after TMZ, volasertib and combined treatment in BT115 cells was measured by Hoechst-Pyronin Y staining (**A**) Reduced level of CD133 expression after treatment in BT115 tumorspheres cells *(***B**) Reduced tumorsphere formation capacity in BT115 cells. The microscopic images (Evos FL Auto, Life Technology) were captured at 10× magnification (**C**,**D**). Significant reduction in the level of side population after combined treatment of volasertib and TMZ in irradiated and non-irradiated BT115 cells. Ordinary one-way ANOVA, SD; [significance at ≤0.001 (***) and 0.12 (ns)] (**E**).

Increased ROS and cell cycle arrest have been shown to induce quiescence in cancer stem cells [24,25]; hence we analyzed G1-G0 transition to identify quiescent cells during cell cycle arrest. We used BT115 cells based on high CD133 positivity and side population, a marker for stem cells as compared to LN229 cells. BT115 showed increased G1-G0 transition after TMZ treatment (16.9%) and radiation (10.2%) compared to the untreated-control cells (4.35%). Volasertib significantly inhibited G1–G0 transition in both non-irradiated (2.58%) and irradiated cells (0.53%) (Figure 3A). In contrast, the combinatorial drug effect was found lower than volasertib alone. We also observed an increased sub-G1 population (Supplementary Figure S2) indicating apoptotic cell death after treatment of volasertib alone and with TMZ combination.

To identify the effect of volasertib on stemness in GBM cells, we measured the level of CD133, the potency of tumorspheres (TS) formation and the level of SP in BT115 cells. Compared to CD133+ expression levels in controls (82.1%), we observed a marked reduction in CD133+ cells after TMZ (39.5%) and volasertib treatment (23.8%) compared to untreated cells (82.1%) while no additional reduction was detected with combined treatment of TMZ and volasertib (24.7%) (Figure 3B). Similarly, the tumorsphere formation was significantly reduced by volasertib treatment compared to untreated cells and TMZ treatment. TMZ treatment itself was able to significantly reduced tumorsphere formation compared to untreated BT115 cells. We did not find any significant inhibitory effect with combined treatment of TMZ and volasertib over volasertib alone treatment (Figure 3C,D).

As glioblastoma stem cells are enriched in the side population, contributing towards therapy resistance [26]. Hence, we measured the level of SP after drug treatment and radiation with their controls for SP identification (Supplementary Figure S3). We found a significantly decreased level of side population after the combined treatment of volasertib and TMZ (80%) compared to the treatment of TMZ (15%) or volasertib (37%) alone. Recent studies showed induction of stemness after radiation [27], we also confirmed that radiation significantly increased the side population level (103%) in BT115 cells. However, despite the radiation-induced increase in side population, volasertib and TMZ combination synergistically decreased the level of side population (92%) compared to TMZ (78%) and volasertib (65%) in irradiated BT115 cells (Figure 3E). Overall data suggest that combined treatment of volasertib and TMZ significantly reduces the SP despite the radiation-induced increase in GBM cells.

### 3.4. Volasertib Inhibits Key Tumor-Promoting Signaling Pathways in GBM Cells

**Figure 4:**
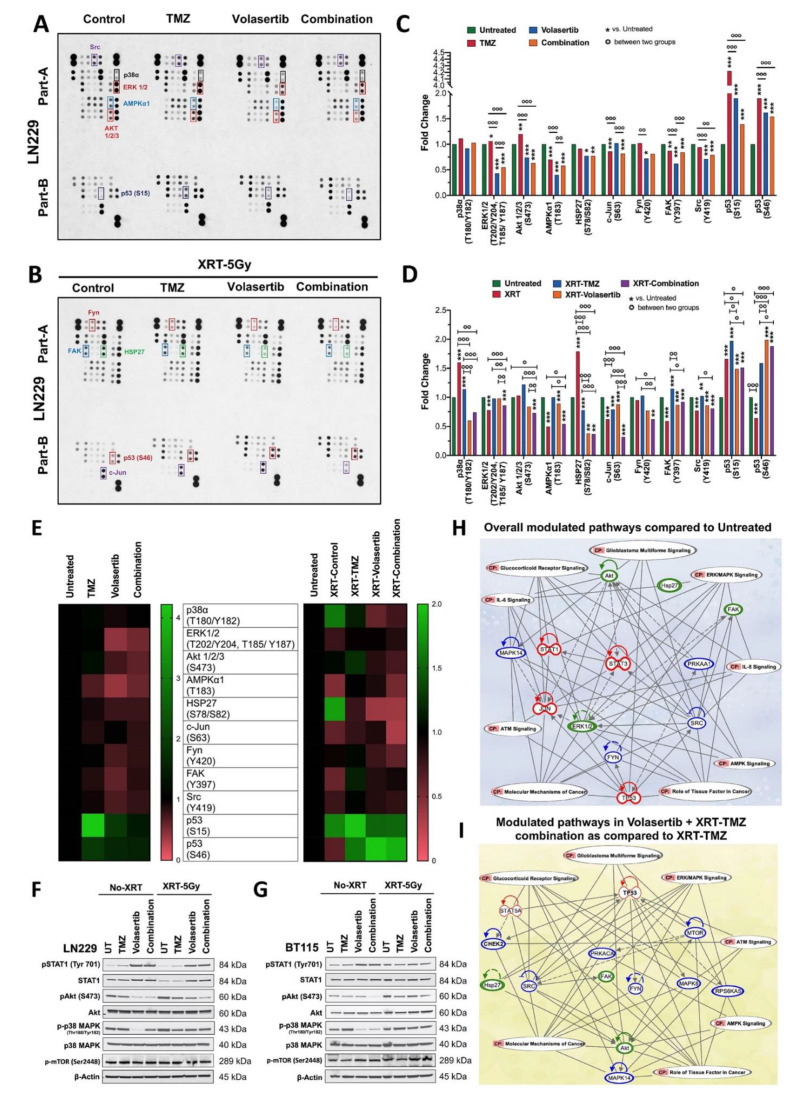
Identification of key signaling modulators. Human Phospho-Kinase profiling in LN229 cells after volasertib and its combination with TMZ in non-irradiated (**A**) and irradiated (XRT-5Gy) (**B**). Fold change difference of significantly altered protein kinases compared to untreated control and individual treatment of volasertib and TMZ in non-irradiated (**C**) and irradiated (XRT-5Gy) (**D**) LN229 cells. Two-way ANOVA with Tukey’s multiple comparison test, error bars; [significance at ≤0.05 (*), ≤0.002 (**), ≤0.001 (***) and 0.12 (ns)]. The heatmap illustration of the fold change of significantly altered protein kinases compared to untreated control and radiation control in volasertib and TMZ treated LN229 cells (**E**) Validation of some key modulated proteins by western blot in LN229 (**F**) and BT115 cells (**G**). The ingenuity pathway analysis (IPA) for a possible mechanism of volasertib and its combination with TMZ and radiation mediated anti-tumor response (**H**). The ingenuity pathway analysis (IPA) for downregulated signaling pathways in Volasertib + XRT-TMZ combination compared to SOC (XRT-TMZ) (**I**). Genes were grouped in specific families by IPA software and color-coded: transcription regulator family (red), kinases (blue) and Others (green). Around the gene, an arrow with a solid line indicates activation, an arrow with a dotted line indicates the regulation of binding and a solid curved line without an arrow denotes the inhibitory status of the gene.

To characterize molecular signaling events regulated by the volasertib alone and with TMZ treatment and radiation in treatment responsive LN229 cells, we performed human phospho-kinase array analysis (Figure 4A,B). Our data suggest the overlapping mechanism of action after treatment of volasertib alone and in combination with TMZ in irradiated and non-irradiated LN229 cells. Broadly, we observed downregulation of phosphorylated ERK1/2, AMPKα1, Akt1/2/3, HSP27, c-jun, Fyn, FAK, Src and upregulation of p53 (S15, S46), in both non-irradiated and irradiated cells (Figure 4C,D). We found higher inhibition of pHSP27, pP38α and c-Jun (S63) in irradiated cells compared to radiation control while non-irradiated cells had higher downregulation of pERK1/2 and increased p53 (S46) compared to untreated cells as shown in heat map analysis (Figure 4E).

To validate selected observations above and test additional pathways, we performed western blot analysis of LN229 and BT115 cells under the various treatment paradigms. Consistent with the array, AKT activation was decreased in both LN229 and BT115 with volasertib alone and/or in combination with TMZ irrespective of radiation treatment. Interestingly, we observed significant higher downregulation of phospho-p38 after volasertib treatment compared to combined treatment in LN229 cells (Figure 4F), however, BT115 cells had significant downregulation of phospho-p38 after both volasertib alone and with TMZ treatment (Figure 4G). We did not find any significant change in phospho-p38 after radiation in both cells. Additionally, we tested Interferon/Stat1 Pathway, a modulator of apoptosis [28] and phospho-mTOR [29] which were not included in the phospho-kinase array for better evaluation of molecular mechanism. We observed an increase in the activation and phosphorylation of STAT1 after volasertib alone and with TMZ in both non-irradiated and irradiated LN229 and BT115 cells. However, no significant change was observed in the expression level of phospho-mTOR irrespective of drug and radiation treatment in both cells (Figure 4F,G). Ingenuity Pathway Analysis (IPA) of the above-mentioned results revealed that differentially modulated kinases after treatment of volasertib alone or in combination with SOC are regulating glucocorticoid receptor, ERK/MAPK, ATM and AMPK signaling pathways in GBM cells cumulatively involved in glioblastoma signaling and molecular mechanism of cancer (Figure 4H,I).

### 3.5. Volasertib Synergizes with TMZ and Radiation to Induce DNA Damage and Modulate DNA Repair Genes Expression in GBM Cells

The activated p53 after volasertib treatment is consistent with previous reports [30,31] and our kinase data suggest modulation of DNA repair capacity after volasertib and combination treatment. Next, we assessed the DNA damage response after the concurrent inhibition of PLK1 with TMZ and radiation, we performed the neutral comet assay after 24 h of treatment and post 24 h of drug release, the latter to assess persistence of DNA damage.

**Figure 5:**
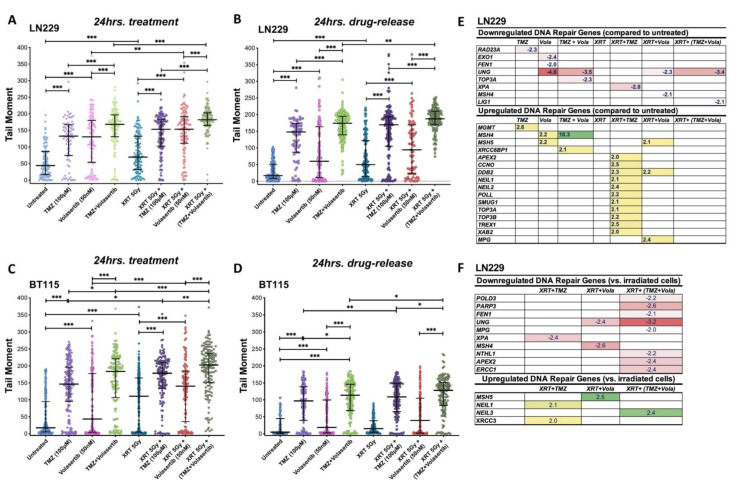
Assessment of DNA damage and altered repair protein during combinatorial drug treatment. DNA damage was measured by neutral comet assay after 24 h of drug treatment in non-irradiated and irradiated LN229 cells (**A**) and BT115 cells (**C**). DNA damage was measured by neutral comet assay after 24 h of drug release 48 h of drug treatment in non-irradiated and irradiated LN229 cells (**B**) and BT115 cells (**D**). Fold regulation of significantly altered repair proteins measured by RT2 profiler in non-irradiated and irradiated LN229 cells as compared to untreated (**E**). Fold regulation of significantly altered repair proteins within irradiated group compared to radiation control (XRT-5GY) (**F**). Ordinary one-way ANOVA, median; [significance at ≤0.05 (*), ≤0.002 (**), ≤0.001 (***) and 0.12 (ns)].

In the LN229 cells, TMZ and volasertib alone produced significant DNA damage which was further potentiated by their combinatorial treatment. After the radiation treatment, we observed an additional significant increase in DNA damage in all treatment groups as compared to radiation control (XRT-5Gy). A significant difference was seen in volasertib alone and combined treatment between non-irradiated and irradiated groups (Figure 5A). We found the persistent level of DNA damage post 24 h of drug release. The level of damage was similar in TMZ and combinatorial treatment but prominently reduced in volasertib treatment in both non-irradiated and irradiated groups (Figure 5B). In BT115 cells, damage by combinatorial treatment was found significant over TMZ and volasertib alone in both non-irradiated and irradiated groups. Radiation treatment further potentiated the effect of all treatment groups (Figure 5C). When the drug was released, the level of DNA damage was reduced but with persistent damage. Here also, the combinatorial treatment showed significant damage compared to their single-agent treatment in both non-irradiated and irradiated groups. However, only TMZ and combinatorial treatment showed significant damage after radiation treatment when compared to non-irradiated cells (Figure 5D).

The persistent level of DNA damage suggested specific mechanisms of generating and/or repairing DNA damage for each treatment regimen. Next, we performed the RT2 profiler PCR array for DNA repair assessment in LN229 cells post 24 h of drug release which provides enough time to repair DNA damage. We observed the upregulation of the MSH4 and MSH5 genes involved in the mammalian meiotic recombination repair [32] after treatment with volasertib and its combination with TMZ and radiation. However, the MSH4 gene was found downregulated by volasertib treatment in irradiated cells. TMZ treatment in irradiated cells showed upregulated repair genes involved in base excision repair (NEIL1, NEIL2, POLL, SMUG1) and single-strand break repair (TOP3A, TOP3B). Volasertib alone and in combination with TMZ downregulated Uracil DNA glycosylase (UNG) which in turn increases DSB, apoptosis and sensitization to genotoxic stress [33] irrespective of radiation treatment (Figure 5E). Other key repair genes such as FEN-1, LIG1, XPA and TOP3A were also found downregulated indicating reduced tumor progression, chemo-resistance, alternative end-joining (Alt-EJ) and nucleotide excision repair (NER) [34,35] (Figure 5E). Within the irradiated group, we found downregulation of UNG, FEN1, PARP3, POLD, NTLH1, ERCC1 and MPG which are linked to increased DSBs, sensitivity to alkylating agents, impaired tumor growth and reduced EMT, DSB repair and mitotic progression [33,34,36,37,38,39] (Figure 5F). Collectively, this robust modulation of DNA repair genes with combinatorial treatment suggests that volasertib may enhance the effectiveness of standard XRT and TMZ in part through potentiation of DNA damage.

### 3.6. Combined Treatment of Volasertib and TMZ Reduces Tumor Growth and Stemness and Increases DNA Damage and Apoptotic Cell Death in Glioblastoma Cells In Vivo

**Figure 6:**
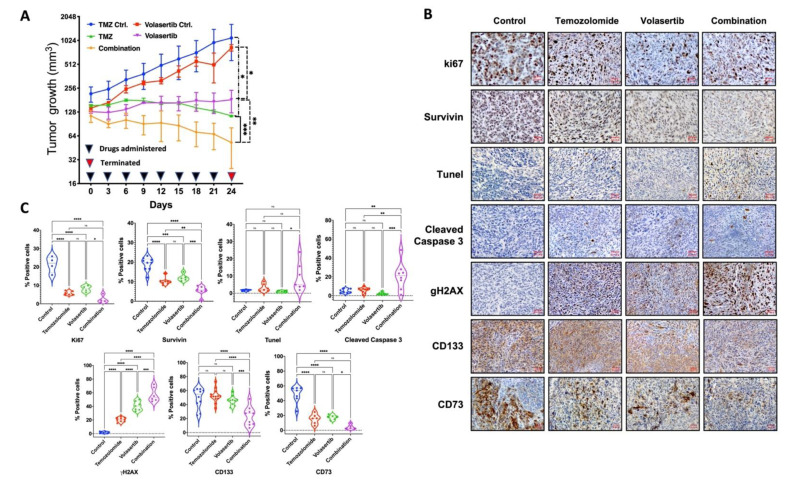
Effect of combinatorial treatment on tumor growth in a mouse xenograft model. Tumor growth was measured every three days after treatment of TMZ (25 mg/kg/3 days), volasertib (10 mg/kg/3 days) and combination LN229 cells xenograft for 24 days. RM one-way ANOVA, with the Geisser-Greenhouse correction and Tukey’s multiple comparison test, error bars, SD; [significance at 0.0332 (*), 0.0021 (**), 0.0002 (***), <0.0001 (****) and 0.1234 (ns)] (**A**). The immunohistochemical staining was performed using Ki67, survivin, tunel, cleaved caspase-3, gH2AX, CD133 and CD73. The image captured at 10× magnification (**B**). Quantification of IHC staining (**C**) by IHC profiler plugin in ImageJ software. Data obtained was present as violin plot with the data point and automatic pairwise comparison was used to check significant level between groups.

To examine the in-vivo effect of volasertib alone and its combination with TMZ, we used the xenograft model with LN229 cells. Tumor growth was monitored after treatment with a sublethal dose of TMZ (25 mg/kg/3 days), volasertib (10 mg/kg/3 days) and their combination for 24 days. The treatments were well tolerated by the mice with no signs of systemic toxicity. We observed a significant decrease in tumor growth after treatment of TMZ or volasertib monotherapy when compared to their respective controls. The combined treatment of volasertib and TMZ was found to be most effective and significantly reduced tumor growth compared to single-agent treatment (Figure 6A). The immunohistochemical analysis of xenograft tumors harvested after 24 days demonstrated a reduced level of cell proliferation markers (Ki67, survivin), induced apoptosis (Tunel, cleaved caspase 3), increased DNA damage (gH2AX), reduced stemness (CD133, survivin) and reduced pro-tumorigenic microenvironment (CD73) in volasertib alone and combination-treated mice tissue sections (Figure 6B). In particular, the apparent increase in gH2AX in tumors treated with combined therapy is consistent with in-vitro observations using the comet assay to detect DNA damage (Figure 5A) while decreased Ki67 and CD133 marker expression correspond to reduced cell proliferation and side population (Figure 6C).

## 4. Discussion

Targeted therapeutic adjuncts to enhance current SOC therapy for GBM are urgently needed to improve the median survivals of 15–16 months with TMZ/XRT and 20 months with the addition of tumor treating fields [3]. PLK1 promotes malignancy through diverse mechanisms and has gained increasing interest as a promising therapeutic target in many cancers including GBM [2,7]. A significantly higher expression of PLK1 has been observed in brain tumor cells than in corresponding normal brain tissue. The high level of PLK1 has been associated with lower disease-free and overall survival rates, and poor prognostic factors for glioma patients (Supplementary Figure S4) [40]. Lee et al. 2012 examined the level of PLK1 in brain tumor-initiating cells (BTICs) isolated from patients who expressed 110–470 times more PLK1 than normal human astrocytes [41]. Another study by Amani et al. 2016 (PMID: 27538997) showed a high level of PLK1 in Diffuse intrinsic pontine gliomas (DIPGs) compared to normal brain cells isolated from the brain stem [42].

Recent studies showed synergistic activity of the PLK1 inhibitor (volasertib) with TMZ [2] or XRT in GBM cells [11]. However, the combined effect of volasertib with clinically relevant concurrent TMZ and XRT has not yet been reported. Therefore, we evaluated the mechanisms and relative efficacy of PLK1 inhibition in the context of TMZ and/or XRT treatments in GBM cell lines in-vitro and the synergistic effect of volasertib and TMZ in-vivo. Interestingly, Higuchi et al. 2018 (PMID: 30217967) confirmed that the 2nd and 3rd generations of PLK1 inhibitor volasertib and GSK461364 R had only modest toxicity to proliferating normal human astrocytes [10]. We showed robust effects of volasertib alone and potentiated the effect of concurrent TMZ and XRT in glioma cells through established and previously unreported, but therapeutically relevant, mechanisms of action. Together these data support further preclinical evaluation of volasertib mediated PLK1 inhibition as an adjunct to current SOC GBM therapy.

In various GBM cell lines, volasertib synergized with TMZ to reduce cell viability in a dose-dependent manner (Figure 1A). This corroborates a prior report where PLK1 inhibition enhanced TMZ mediated glioma cell cytotoxicity in-vitro and reduced tumor growth in-vivo [2]. Other studies reported synergistic effects of radiation with volasertib on cell survival through mitotic catastrophe, however, the combined effects of TMZ and volasertib with radiation were not evaluated [9,11]. In our study, volasertib alone had robust cytotoxic effects and in combination, accentuated the cytotoxicity of TMZ and/or XRT and an overall reduction in cell survival (Figure 1B,G), suggesting its potential benefit as an adjunct to SOC.

Along with the pro-apoptotic effect of volasertib, additional experiments revealed new mechanistic insights into volasertib activity in glioma. For example, compared with TMZ or XRT, volasertib alone markedly altered MtMP (high and low) from baseline. When combined with XRT, induced a massive increase in high MtMP levels and the corresponding increase in ROS (Figure 2A–C). These novel observations have therapeutic relevance and are consistent with the known relationship between higher and impaired MtMP with ROS induction and cell death [21,43].

This study also established the combined effects of volasertib with TMZ and/or XRT on glioma stem cell phenotypes. In accord with Liu N et al. 2018, we observed reduced CD133+ cells and sphere formation with volasertib alone [2] which was greater than with TMZ alone, although not further reduced when combined with TMZ (Figure 3B). The inhibition of stem-like cell phenotypes was supported by side population (SP) analysis [26] where volasertib combined with TMZ/XRT generated the most robust reduction in SP even with an XRT mediated induction of SP (Figure 3E) [27]. Volasertib alone and in combination with TMZ and/or XRT had a profound effect on reducing G1-G0 transition which was markedly increased after monotherapies of TMZ and XRT (Figure 3A) [44,45]. The data suggest the intriguing mechanistic possibility through the dual effects by inhibiting quiescence through G1-G0 transition and promoting subsequent G2/M arrest that triggers apoptosis. The increased sub-G1 population after volasertib treatment (Supplementary Figure S1) is also consistent with reduced quiescence and increased cell death [46].

Volasertib anti-tumor effects were further highlighted by an analysis of global signaling. Compared with untreated controls, volasertib treatment paradigms in LN229 altered activity of multiple proteins including reduced pP38α, ERK1/2, Akt1, AMPKα, HSP27, c-Jun, Fyn, FAK, Src and increased in p53 and Stat1 (Figure 4). IPA analysis of the integrated data indicated that volasertib modulates glioma relevant pathways including glucocorticoid receptor [47], AMPK [48], ATM [36] and ERK/MAPK [29] (Figure 4H). Downregulation of c-JUN, HSP27, AMPK, Akt, Fyn and pP38α signaling was unique to cells treated with volasertib plus XRT-TMZ (SOC) (Figure 4I). Reports suggest that AMPK is essential for glioma stem cell survival [48], HSP27 downregulation promotes glioma cell apoptosis, and [49] and c-JUN promotes glioma cell proliferation [50]. Stat1, a modulator of genotoxic responses, implicated in glioma stem cell survival [51] was also activated by volasertib mediated treatments in both LN229 and BT115 (Figure 4 F,G) and concert with p53 may interact to enhance DNA damage and apoptosis [52]. P53 status and activation of ATM, both identified here, are linked to PLK1 function and drug responses. In summary, identified signaling was unique to volasertib mediated anti-tumor response in glioblastoma and provide additional support for the PLK1 inhibition as an adjunct to SOC in glioma and the search for biomarkers that predict its efficacy.

Our pathway analysis and previous reports [8,9,11] indicate PLK1 mediated modulation of DNA damage response. We observed acute DNA damage after volasertib treatment alone and in combination with TMZ, which was further potentiated by XRT (Figure 5A,C). Persistence DNA damage after drug release (Figure 5B,D) was accompanied by altered expression of DNA repair gene (Figure 5E,F). The increased detection of gH2AX in tissue sections from tumors treated with both drugs provided in-vivo corroboration of their combined effects on DNA damage in-vitro (Figure 6B). The levels of acute and persistent DNA damage also correlated with reduced cell survival in both GBM lines (Figure 1F,G). The gene expression data suggest that BER and post-replicative or meiotic recombination damage repair genes [32] were upregulated in irradiated and non-irradiated cells as a compensatory DNA repair system. By contrast, all downregulated repair genes have reported roles in increasing DSB, sensitizing to alkylating agents, impaired tumor growth, reduced EMT, DSB repair and mitotic progression [33,34,36,37,38,39,53]. In total, volasertib alone and combination treatment increased apoptosis by reducing DNA repair capacity and inducing persistent DNA damage. Strikingly, while persistent DNA damage in cells treated with volasertib alone was lower than TMZ alone (Figure 5B,D), its addition to SOC dramatically abrogated the upregulated of DNA repair genes (Figure 5E) while downregulating other DNA repair genes with the persistent level of DNA damage. Collectively, these data suggest that volasertib may play a key role in promoting DNA damage through coordinated cell cycle deregulation and inhibition of DNA damage repair.

**Figure 7:**
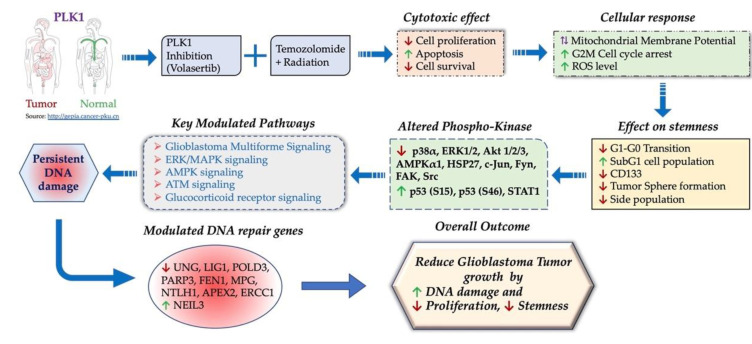
A graphical abstract for the combinatorial effect of PLK1 inhibition with the concurrent temozolomide and radiation treatment.

As noted in results, cell-line-dependent differences were evident in some responses to volasertib alone or combination with TMZ/XRT. For example, differential readouts were observed for apoptosis, (Figure 1B,D), ROS (Figure 2B,C), comet assay assessed DNA damage and cell survival (Figure 5A–F) in BT115 and LN229 cells. These differences underscore some potential limitations of the present study. These differential responses may reflect mechanisms related to distinct molecular phenotypes and differences in proliferation rate, cellular signaling and elevated stemness with altered DNA repair machinery in each cell line. Increasing the time of drug and radiation exposure in BT115 could potentiate its combination effect but was not altered in this study to maintain experimental uniformity between cell lines and assays. This can be resolved through future analysis of the comparative response and molecular analysis in larger sample sizes. Due to the different mechanisms of action for volasertib (cell cycle inhibitor with DNA repair machinery interactions) and TMZ (alkylating-based induction of DNA damage) differences in experimental readouts with combinatorial treatment may vary depending on experimental theme and treatment exposure. Furthermore, in many assays, the proportionally large effects of volasertib alone may have masked the potential degree of synergistic potential in combination with TMZ and/or XRT. Therefore, future investigations will be directed to evaluate a broader range of dose combinations as well as sequencing of drug and XRT delivery in additional cell lines. Further, disparities between cell death and clonogenic survival in response to TMZ or volasertib alone observed in BT115 (Figure 1E and Figure 5F) suggest a mechanism unique to BT115 that drives differential responses to more acute drug exposure versus those driving subsequent growth after selection in longer-term clonogenic survival assays. Regardless of potential limitations, we believe that the differences noted above more likely reflect cell line-specific and assay-specific effects rather than experimental variations. Further, the data collectively indicated that volasertib acts through mechanisms that are in part unique from those of TMZ and/or radiation, and based on multiple complementary and unique functional effects, supported the potential role of volasertib as a targeted therapeutic adjunct to standard TMZ/XRT therapy (Figure 7).

Using multiple orthogonal in-vitro cytotoxicity and functional/mechanistic assays, our data supported the potential value of volasertib as an adjunct to SOC. Multiple mechanisms were altered by volasertib and future studies will be directed towards understanding their relative contributions to promote cell death and overcome resistance. Of interest, Dong et al. (2018) reported that volasertib monotherapy had no significant anti-tumor activity in-vivo but synergized with XRT to inhibit tumor growth and prolong animal survival [11]. Testing the effects of volasertib and TMZ in the context of radiation would have been desirable. Due to concerns over toxicity and potential lethality with the large radiation fields required to treat the xenografts, we chose to use the in-vivo platform to confirm the additive effects of TMZ and volasertib observed in-vitro. The current in-vivo data supports the potential relevance of volasertib as a therapeutic adjunct through marked inhibition of tumor growth alone and in conjunction with TMZ and corresponding decreases in expression of stem cell markers (survivin and CD133) and increased detection of DNA damage (gH2AX) and apoptosis (tunel, cleaved caspase-3). With the advent of more precise radiation delivery systems that mitigate toxicity, the impacts of radiation are planned for future studies. In agreement with our study, Higuchi et al. (2018) showed a reduction in tumor growth in the xenograft model of MMR-deficient-TMZ resistant glioblastoma but also reported disparate activities of volasertib (and other PLK1 inhibitors) in xenograft versus orthotopic models suggesting that BBB penetration may be a therapeutic impediment [10]. Therefore, current data support the therapeutic potential of volasertib alone or as a therapeutic adjunct to SOC in GBM.

## 5. Conclusions

The current study highlights the effects of volasertib in GBM and provides new mechanistic insights into volasertib-mediated PLK1 inhibition and potentiation of current GBM SOC (Figure 7). These findings postulate rationale for future pre-clinical studies to establish treatment paradigms with PLK1 inhibition-based therapy for possible translation to human trials.

## Data Availability

The date presented in the study are available in: http://gepia.cancer-pku.cn/detail.php?gene=PLK1 (accessed on 22 September 2021).

## Supplementary Materials

The following are available online at https://www.mdpi.com/article/10.3390/cancers13205114/s1, Figure S1: Clonogenic cell survival analysis: (A) significant reduction in clonogenicity after combined treatment of volasertib and TMZ in irradiated and non-irradiated LN227 cells. (B) significant reduction in clonogenicity after combined treatment of volasertib and TMZ in irradiated and non-irradiated BT115 cells. Figure S2: Cell cycle transition. SubG1 population of cell cycle measured by Hoechst-Pyronin Y staining in BT155 cells. Serum starved cells were used as an experimental control group. Figure S3: The level of side population after volasertib and combination-treated BT115 cells: (A) significant reduction in side population after combined treatment of volasertib and TMZ in irradiated and non-irradiated BT115 cells. Fumitremorgin C (FTC) treatment was used for the identification of the side population. Figure S4: PLK1 expression and overall survival in glioblastoma. Figure S5: Original figures of Phospho-kinase assay and western blot for Figure 4. Supplementary Methodology: Cell viability (MTT assay) and Neutral Comet assay. Table S1: List of antibodies used in the study.
