# Supplementary material for: Combinatorial Effect of PLK1 Inhibition with Temozolomide and Radiation in Glioblastoma
Source: Cancers (Basel). 2021 Oct 12;13(20):5114. doi: 10.3390/cancers13205114 (PMC8533781; doi:10.3390/cancers13205114)
Supplement: Supplementary file 1 [file cancers-13-05114-s001.zip › cancers-1387045-supplementary.pdf]

Combinatorial Effect of PLK1 Inhibition with Temozolomide and Radiation in Glioblastoma

Arvind Pandey, Satyendra C. Tripathi, Junhua Mai, Samir M. Hanash, Haifa Shen, Sankar Mitra and Robert C. Rostomily

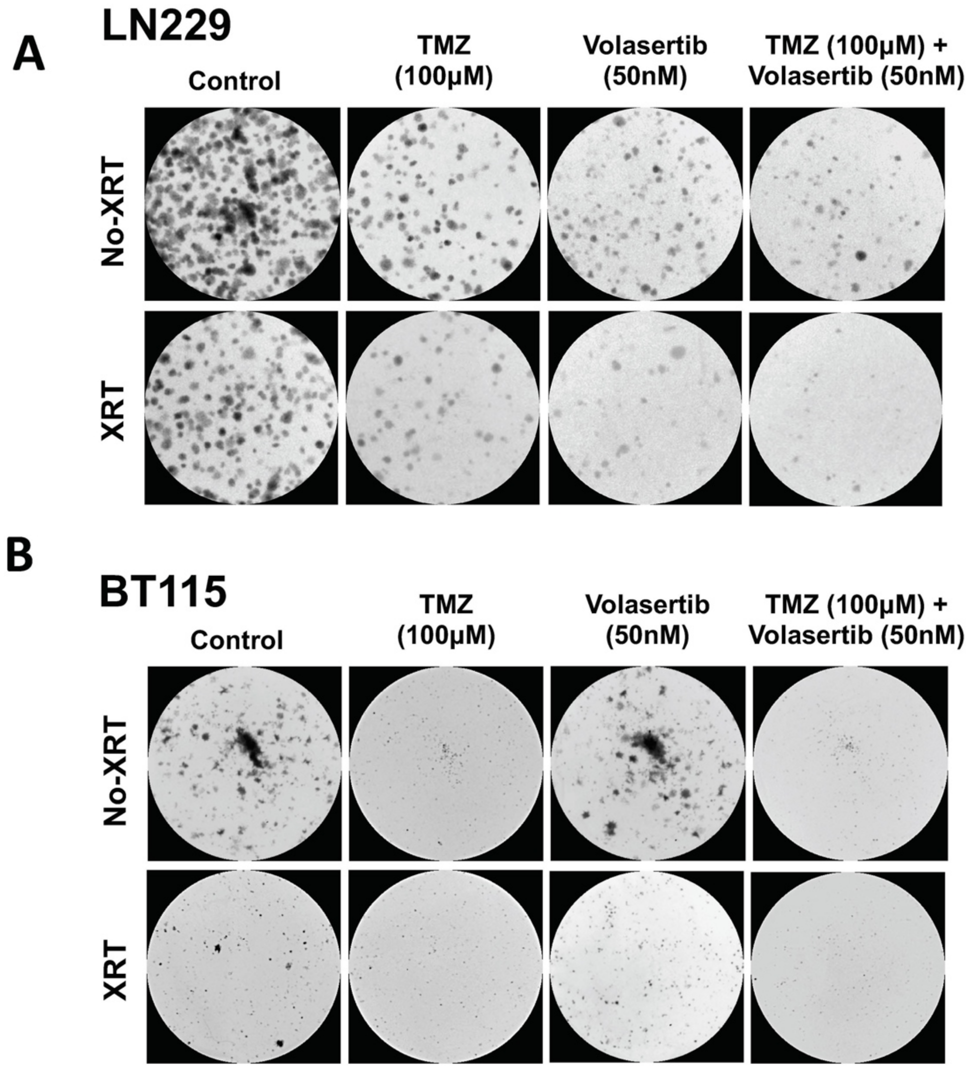

Supplementary Figure S1: Clonogenic cell survival analysis: (A) significant reduction in clonogenicity after combined treatment of volasertib and TMZ in irradiated and non-irradiated LN227 cells. (B) significant reduction in clonogenicity after combined treatment of volasertib and TMZ in irradiated and non-irradiated BT115 cells.

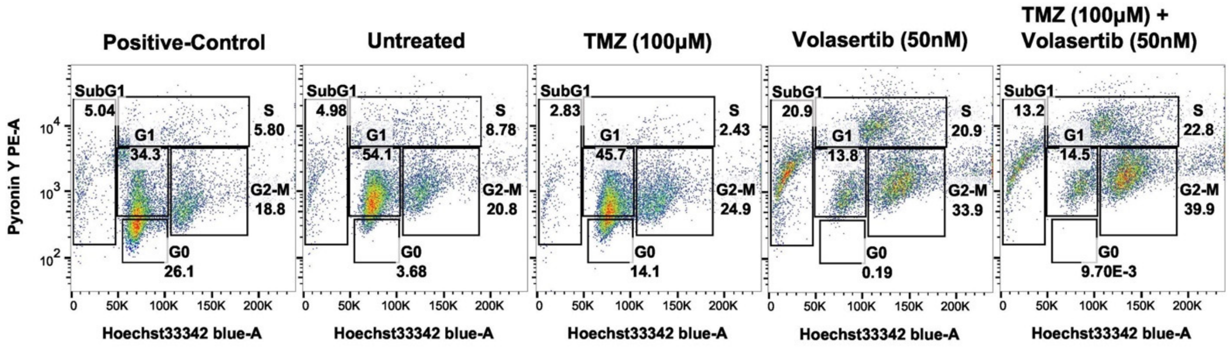

Supplementary Figure S2: Cell cycle transition. SubG1 population of cell cycle measured by Hoechst-Pyronin Y staining in BT155 cells. Serum starved cells were used as experimental control group.

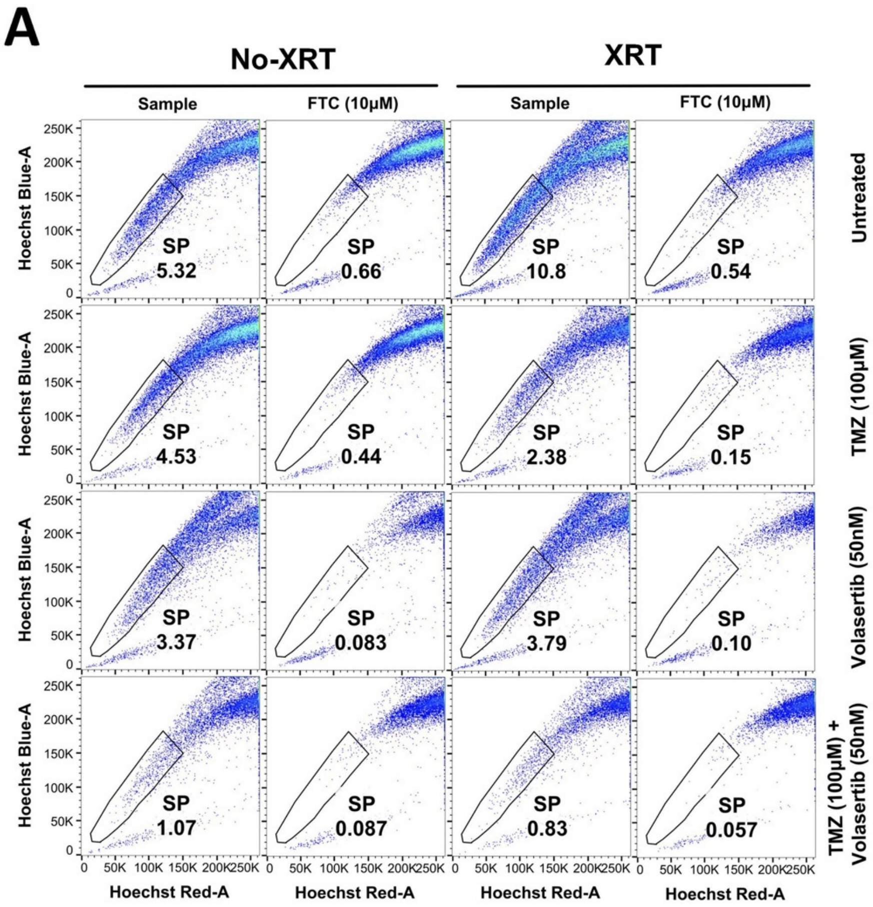

Supplementary Figure S3: The level of side population after volasertib and combination treated BT115 cells: (A) significant reduction in side population after combined treatment of volasertib and TMZ in irradiated and non-irradiated BT115 cells. Fumitremorgin C (FTC) treatment was used for identification of side population.

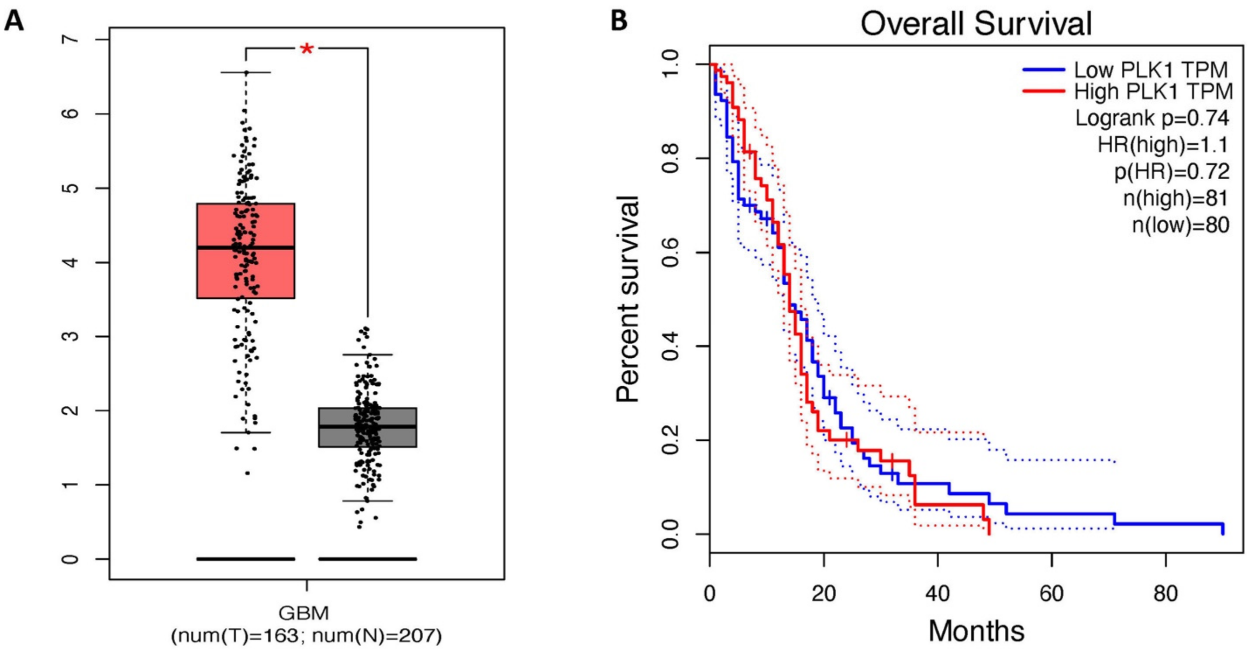

Supplementary Figure S4: PLK1 expression and overall survival in glioblastoma. (A) A fold change expression of PLK1 in tumor (red color) vs normal cells (grey color) analyzed with matched TCGA normal and GTEx data. (B) Overall median survival with high vs low PLK1 level in glioblastoma patients. Source: http://gepia.cancer-pku.cn/detail.php?gene=PLK1

A

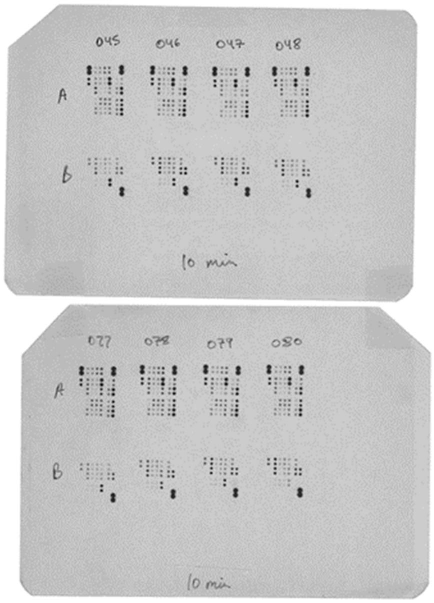

Human Phosphokinase array

B

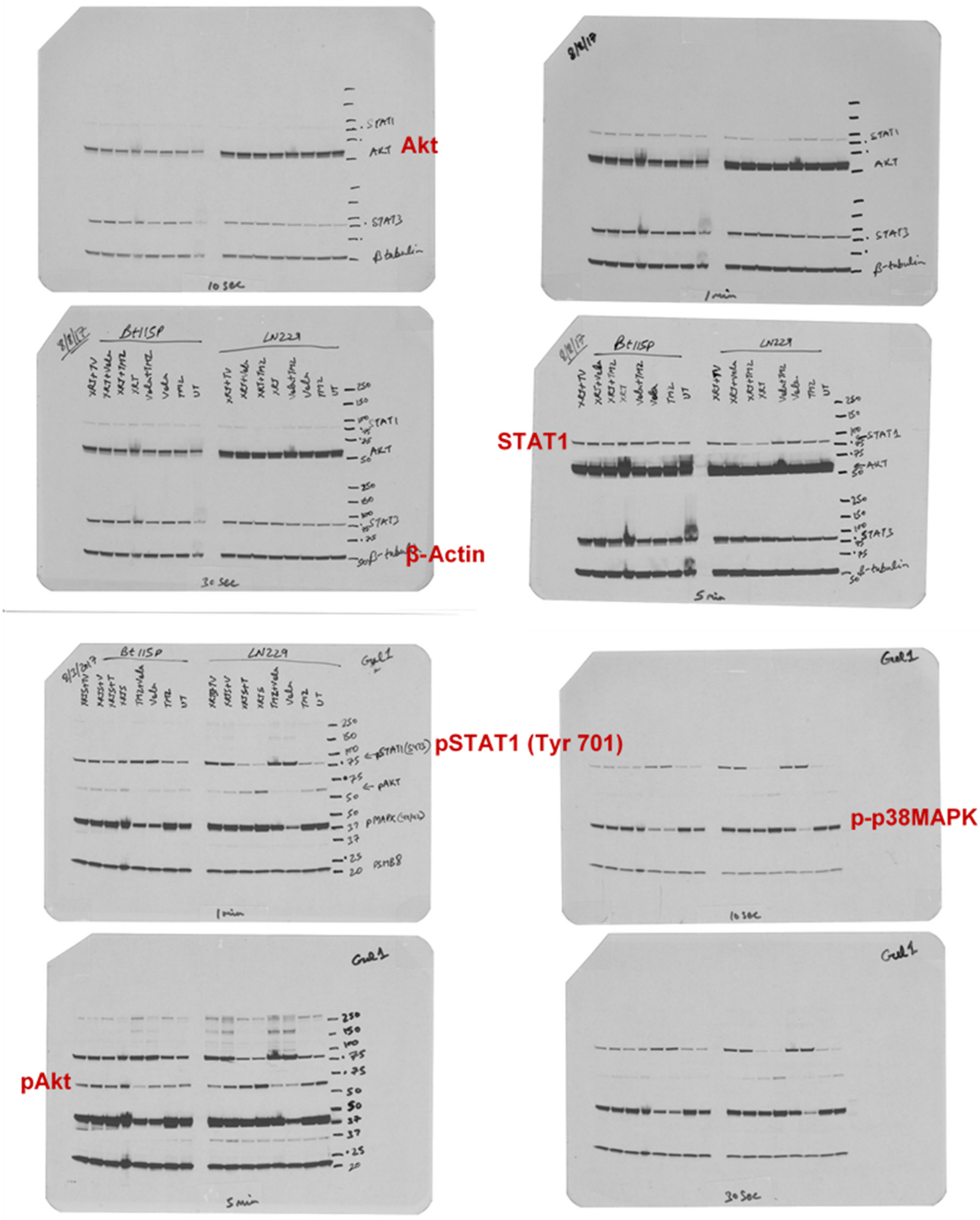

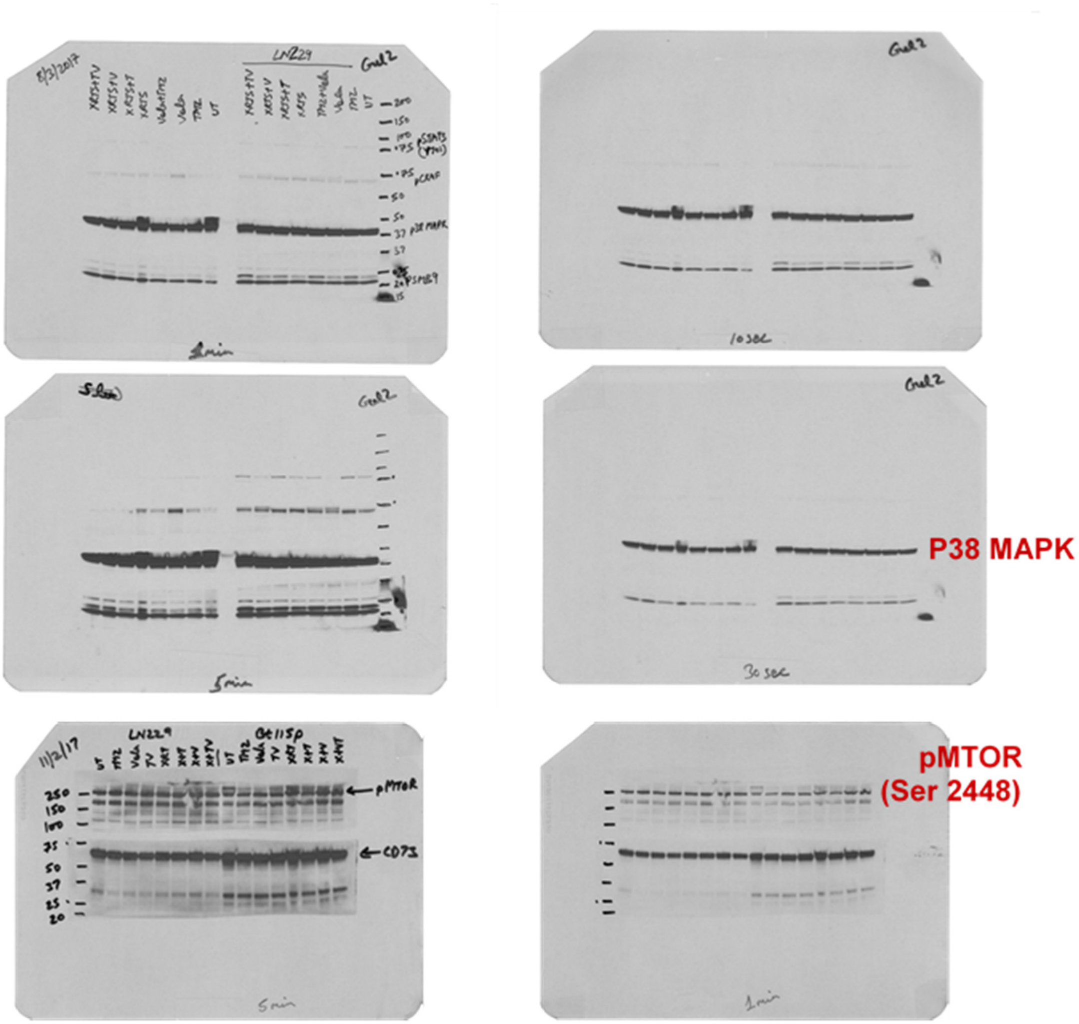

Supplementary Figure S5 (**A**) Original Figures of Western Blot Image for Figure 4 A. (**B**) Original Figures of Western Blot Image for Figure 4 F-G.

Supplementary Table S1.

List of Antibody used in the study

Antibody	Company	Catlouge No.	Dilution	Application
CD133	Miltenyi Biotec	130-113-108	1:50	Flow Cytometry
Stat1 (D1K9Y)	Cell Signaling	14994	1:1000	Western Blotting
Phospho-Stat1 (Tyr701)	Cell Signaling	9167	1:1000	Western Blotting
Akt	Cell Signaling	9272	1:1000	Western Blotting
Phospho-Akt (Ser473)	Cell Signaling	4060	1:2000	Western Blotting
p38α MAPK (L53F8)	Cell Signaling	9228	1:1000	Western Blotting
Phospho-p38 MAPK (Thr180/Tyr182)	Cell Signaling	4511	1:1000	Western Blotting
Phospho-mTOR (Ser2448)	Cell Signaling	5536	1:1000	Western Blotting
β-Actin (8H10D10)	Cell Signaling	3700	1:1000	Western Blotting
Ki67	Abcam	ab15580	2 µg/ml	IHC-P

Survivin (71G4B7)	Cell Signaling	2808	1:400	IHC-P
Cleaved Caspase-3 (Asp175)	Cell Signaling	9661	1:400	IHC-P
gamma H2A.X (phospho S139)	Abcam	ab81299	1:1000	IHC-P
CD133	Abcam	ab19898	1:400	IHC-P
NT5E/CD73 (D7F9A)	Cell Signaling	13160	1:200	IHC-P

Supplementary Methodology:

Cell viability (MTT assay)

Volasertib and temozolomide were purchased from Selleckchem. The cytotoxic effects of volasertib (nM) alone and with TMZ (μM) in GBM cells were determined by MTT assay as described earlier (13). Briefly, the cells were incubated in triplicate in a 96-well plate and treated with volasertib alone (nM) and with TMZ (μM) at various doses (50 to 800) in a final volume of 0.1 ml for 24h at 37°C in a CO2 incubator. Thereafter, 0.025 ml of MTT solution (5 mg/ml in PBS) was added to each well. After 2h incubation at 37°C, the lysis buffer was added, and the extract was incubated overnight at 37°C for solubilization of formazan crystals. The OD at 570 nm was measured using a 96-well multi-scanner auto-reader (Biotek, Winooski, VT) with the lysis buffer serving as the blank.

Neutral Comet assay

The Comet analysis of DNA double-strand breaks was carried out at neutral pH using Trevigen comet assay kit (Cat: 4250-050-K) following the manufacturer's protocol. Samples were visualized at 10X under microscope (Evos FL Auto, Life Technology). To quantify the DNA damage tail length (TL) and tail moment (TM) were evaluated. The tail length (length of DNA migration) is related directly to the DNA fragment size. The tail moment was calculated as the product of the tail length and the fraction of DNA in the comet tail. At least 50 randomly selected cells were scored per sample. Image was analyzed by Open Comet plugin in ImageJ for various comet parameters (18).

Reference:

13. Pandey A, Vishnoi K, Mahata S, Tripathi SC, Misra SP, Misra V, et al. Berberine and Curcumin Target Survivin and STAT3 in Gastric Cancer Cells and Synergize Actions of Standard Chemotherapeutic 5-Fluorouracil. Nutr Cancer 2015;67(8):1293-304 doi 10.1080/01635581.2015.1085581.

18. Gyori BM, Venkatachalam G, Thiagarajan PS, Hsu D, Clement MV. OpenComet: an automated tool for comet assay image analysis. Redox Biol 2014;2:457-65 doi 10.1016/j.redox.2013.12.020.
